# Incidence, Associated Risk Factors, and Outcomes of Postoperative Arrhythmia After Upper Gastrointestinal Surgery

**DOI:** 10.1001/jamanetworkopen.2022.23225

**Published:** 2022-07-21

**Authors:** Felix Rühlmann, Tobias Tichelbäcker, Alma Franziska Mackert, Deborah Engelhardt, Andreas Leha, Markus Bernhardt, Michael Ghadimi, Thorsten Perl, Azadeh Azizian, Jochen Gaedcke

**Affiliations:** 1 Department of General, Visceral, and Pediatric Surgery, University Medical Center Göttingen, Göttingen, Germany; 2 Clinic III for Internal Medicine, Heart Center of University Hospital of Cologne, Cologne, Germany; 3 Institute of Medical Statistics, University Medical Center Göttingen, Göttingen, Germany

## Abstract

**Importance:**

New-onset postoperative arrhythmia, which most often presents as postoperative atrial fibrillation (AF), is a frequent complication in patients undergoing visceral surgery of the upper gastrointestinal tract. Its relevance for patients’ outcomes is unknown.

**Objective:**

To assess the incidence of arrhythmia after upper gastrointestinal surgery, its risk factors, and its short- and long-term implications for patient outcomes.

**Design, Setting, and Participants:**

This cohort study included 1210 patients who underwent surgery of the upper gastrointestinal tract (esophagus, stomach, or pancreas) at the University Medical Center Göttingen in Germany between January 2012 and December 2018. Follow-up was performed between February and May 2020. Patients were excluded if they had a preexisting cardiac arrhythmia or pacemaker.

**Main Outcomes and Measures:**

The incidence of atrial fibrillation (AF) was recorded in most cases of postoperative arrhythmia; therefore, the analysis focused on postoperative AF. A multivariable logistic regression model was used to assess associations between surgical complications and postoperative AF occurrence, with odds ratios and 95% CIs reported.

**Results:**

A total of 1210 patients (median [IQR] age, 62 [19-90] years; 704 [58.2%] men) were enrolled in this study. Postoperative arrhythmia was recorded in 100 patients (8.3%). Among the different procedures, esophagectomy was associated with the highest incidence of postoperative AF (45.5% in complex esophageal resections and 17.1% in elective thoracoabdominal esophagectomies). The incidence of postoperative AF was associated with prolonged length of stay in the intensive care unit (23.4 days for patients with postoperative AF vs 5.9 days for those without; *P* < .001). Four factors were associated with the occurrence of postoperative AF: patients’ age (OR, 1.06; 95% CI, 1.03-1.08; *P* < .001), intraoperative surgical complications (OR, 2.47; 95% CI, 1.29-4.74; *P* = .006), infections (OR, 2.23; 95% CI, 1.31-3.80; *P* = .003), and organ failure (OR, 4.01; 95% CI, 2.31-6.99; *P* < .001). In the multivariable analysis, postoperative AF (OR, 7.08; 95% CI, 2.75-18.23; *P* < .001) and sepsis (OR, 10.98; 95% CI, 3.91-30.81; *P* < .001) were associated with in-hospital mortality. At a median 19-month follow-up, 20 of 74 patients (27.0%) with postoperative AF developed recurring episodes of arrhythmia after discharge.

**Conclusions and Relevance:**

This cohort study found that the postoperative AF was associated with an increased length of stay in the intensive care unit and in-hospital mortality in patients after upper gastrointestinal tract surgery. In addition, postoperative AF was associated with development of permanent or paroxysmal arrhythmia after discharge.

## Key Points

**Question:**

What are the incidence of, risk factors for, and outcomes associated with arrhythmia after upper gastrointestinal tract surgery?

**Findings:**

In this cohort study of 1210 patients, the incidence of postoperative arrhythmia was 8%, with substantial differences in incidence found among different upper gastrointestinal tract procedures. Postoperative atrial fibrillation was associated with increased in-hospital mortality, prolonged intensive care unit stay, and development of permanent or paroxysmal atrial fibrillation after discharge.

**Meaning:**

Results of this study suggest that postoperative atrial fibrillation is associated with severe postoperative complications.

## Teaser

This cohort study examines the incidence of developing arrhythmia after upper gastrointestinal tract surgery as well as the risk factors for and outcomes of postoperative arrhythmia.

## Introduction

Surgical feasibility is increasingly being extended by advanced surgical techniques, safe anesthesiologic procedures, and innovative intensive care. Modern medicine therefore enables surgical treatment for an increasing subset of patients, including those who are older, have multimorbidity, or have oncological advanced disease. However, this progressive process needs to be accompanied by careful perioperative treatment for patients with different preexisting organ deficiencies and risk factors. New-onset postoperative arrhythmia, particularly postoperative atrial fibrillation (AF), is a frequent complication in the immediate postoperative period and represents a relevant clinical issue.^1,2^

In cardiac surgery, postoperative AF has been well analyzed: the incidence of postoperative AF ranges between 20% and 50%^3,4,5^ and is associated with a 4- to 5-fold risk of recurrence of AF within the following 5 years.^6,7^ In addition, postoperative AF is a relevant risk factor for thromboembolic stroke, myocardial infarction, and death.^8,9,10^ Additional complications include infections, bleeding, kidney complications, hemodynamic instability, prolonged hospital stay, and increased in-hospital mortality.^2,11,12^

The detection of postoperative AF in intensive care units (ICUs) is feasible. Because most patients are monitored with continuous electrocardiography (ECG), the onset of postoperative AF can be ascertained unequivocally, and rate control (resting heart rate <100 beats per minute) as well as rhythm control (restoring sinus rhythm) within 48 hours after onset of postoperative AF are potential strategies that depend on the patient’s hemodynamic stability and symptoms. Regardless of the strategy chosen, systemic anticoagulation should be established in the acute clinical setting.^13^

Although the occurrence and management of postoperative AF after cardiac surgery are well documented, its incidence after noncardiac surgery has not been well analyzed.^14^ Knowledge of the incidence of postoperative AF and its long-term outcomes after visceral surgery would raise awareness of this issue and may prompt adjustment of the perioperative treatment of patients to improve safety. Although some studies have analyzed the incidence of postoperative AF after esophagectomy,^15,16,17,18,19^ only a few have examined other types of visceral surgery.^20,21^

In this study, we aimed to (1) ascertain the incidence of postoperative AF in the ICU after visceral surgery by retrospectively analyzing data from patients who underwent surgical procedures of the upper gastrointestinal tract, (2) identify possible risk factors for postoperative AF, and (3) investigate the immediate and long-term outcomes of postoperative AF.

## Methods

### Study Cohort and Variables

In this cohort study, we screened all patients (n = 1418) who underwent surgery of the upper gastrointestinal tract and were monitored in the ICU at the University Medical Center Göttingen, Göttingen, Germany, from January 1, 2012, to December 31, 2018. Follow-up was performed between February and May 2020. The surgical procedures included at least 1 of the following: esophagectomy (extended, total, or partial), gastrectomy, gastric sleeve resections, pylorus-preserving pancreaticoduodenectomy, Whipple procedure, pancreatectomy (distal or total), and multivisceral resections (resections of ≥2 organs that are not usually resected simultaneously; resection of the gallbladder and appendix were not included). The patients were screened for preexisting cardiac arrhythmia diagnosis and a pacemaker. Furthermore, the ECG that is normally performed before surgery was screened for existing arrhythmia. Cardiac arrhythmia included AF and atrial flutter, supraventricular tachycardia (atrioventricular reentrant tachycardia, atrioventricular nodal reentry tachycardia), ventricular tachycardia, sinus arrhythmias, and atrioventricular block (second and third degree). All patients with preexisting cardiac arrhythmia or pacemaker were excluded from further analysis (n = 208). Thus, 1210 patients were enrolled in this retrospective study (eFigure 1 in the Supplement). Approval for the study was obtained from the ethics committee at the University Medical Center Göttingen, and patients provided written informed consent. We followed the Strengthening the Reporting of Observational Studies in Epidemiology (STROBE) reporting guideline.

All patients were observed with an ECG monitor device during and after surgery. Observation with an ECG monitor device in the wards was performed for at least 18 hours postoperatively. Monitoring started with induction of anesthesia and stopped with discharge from the ICU or intermediate care unit. Atrial fibrillation was confirmed by ECG and interpreted by experienced intensive care physicians. Atrial fibrillation was defined using the European Society of Cardiology guidelines as the minimum duration of an ECG tracing of AF required to establish the diagnosis of clinical AF of at least 30 seconds.^13^ Electrocardiographic characteristics of AF include irregular R-R intervals, absence of distinct repeating P waves, and irregular atrial activations. Automatic reports were not used for the study. A 12-lead ECG was performed regularly as confirmation if AF was detected on the ECG monitor device. Retrospectively, we used the documented *International Statistical Classification of Diseases and Related Health Problems, Tenth Revision* codes to identify patients in the ICU who had specific arrhythmia, including AF and atrial flutter, supraventricular tachycardia, ventricular tachycardia, sinus arrhythmia, and atrioventricular block.

All patients’ data were screened for a set of preexisting cardiovascular diseases (hypertension, diabetes, coronary heart disease, peripheral artery occlusive disease, myocardial infarction, heart failure, valvular heart disease, thrombotic or embolic event, angiopathy, and coagulopathy). We also screened for previously performed cardiac treatments, including interventional and surgical procedures.

Furthermore, we screened for surgical complications, including anastomosis insufficiency, wound healing deficit or burst abdomen, chyle fistula, bile leakage, pancreatic fistula, other fistulae or abscess, and abdominal compartment syndrome as well as the need for a second surgery because of a postoperative complication. Also, nonsurgical complications, including myocardial infarction, thrombosis, electrolyte disorders, infections (including pneumonia), sepsis, and organ failure, were analyzed. All these parameters were assessed to preselect complications as independent variables for the multivariable logistic regression model.

For all patients who developed postoperative AF, we conducted follow-up screening for permanent arrhythmia, thromboembolic events, and overall survival. Patients, their family doctors, and, if available, their cardiologists were contacted. A standardized survey was used to identify the patient’s surgical and medical history (eFigure 2 in the Supplement).

### Statistical Analysis

Comparisons were performed using the Mann-Whitney *U* test for continuous variables and the χ^2^ test or Fisher exact test for categorical variables. A multivariable logistic regression model was developed using the 10:1 rule^22^ to confirm the independent association between postoperative surgical complications and postoperative AF occurrence. Preselected surgical complications were set as independent variables, whereas the occurrence of postoperative AF was set as a dependable variable. In the second multivariable logistic analysis, surgical complications were again set as independent variables, whereas in-hospital mortality was set as the dependent variable. Odds ratios (ORs) are presented with 95% CIs.

Surgical complications were selected as promoting factors for these models using single tests in advance. All selected variables met the following conditions: association with the occurrence of postoperative AF (*P* < .05) and 20 or more patients in each group. In addition, all selected independent variables in the second multivariable logistic regression model were set to 3 (10% of 30), because 30 patients died during hospitalization. All selected variables met the following conditions: association with the occurrence of death during hospitalization (*P* < .05) and 10 or more patients in each group.

Statistical analyses were performed using R software, version 4.0.2 (R Foundation for Statistical Computing). The global significance level was set at α = .05. The threshold for statistical significance was a 2-sided *P* < .05.

## Results

### Demographic Data

A total of 1210 patients (median [IQR] age, 62 [19-90] years; 704 [58.2%] men and 506 [41.8%] women) who underwent upper gastrointestinal tract surgery were included in the cohort. Surgical procedures included 228 (18.8%) elective thoracoabdominal esophagectomies, 11 (0.9%) emergency esophagectomies, 6 (0.5%) smaller esophageal resections, 96 (7.9%) gastrectomies, 105 (8.7%) extended gastrectomies, 77 (6.4%) partial gastric resections, 157 (13.0%) gastric sleeve resections or gastric bypass surgeries, 357 (29.5%) pylorus-preserving pancreaticoduodenectomies or Whipple procedures, 78 (6.4%) distal pancreatectomies, 24 (2.0%) total pancreatectomies, and 71 (5.9%) multivisceral resections.

A total of 812 patients (67.1%) were diagnosed with malignant neoplasia. Three hundred forty-six patients (28.6%) had 2 or more preexisting cardiac impairments and risk factors, such as hypertension, diabetes, coronary heart disease, peripheral artery occlusive disease, myocardial infarction, heart failure, valvular heart disease, thrombotic or embolic events, angiopathy, or coagulopathy, whereas 100 patients (8.3%) had previously undergone interventional or surgical cardiac treatment. eTable 1 in the Supplement outlines all demographic and clinical data of the enrolled patients.

### Incidence of Postoperative AF and Time Span Between Surgery and Postoperative AF

Postoperative arrhythmia was recorded in 100 patients (8.3%). In 99 of these patients (99.0%), AF was the most common type of arrhythmia detected and sometimes was found in combination with other forms of arrhythmia. We recorded tachycardic AF (n = 83 [83.0%]), normofrequent AF (n = 16 [16.0%]), asystole (n = 12 [12.0%]), ventricular fibrillation (n = 5 [5.0%]), atrial flutter (n = 5 [5.0%]), second- and third-degree atrioventricular blocks (n = 3 [3.0%]), arrhythmia with intraoperative cardiopulmonary resuscitation (n = 2 [2.0%]), bradycardic AF (n = 1 [1.0%]), and ventricular tachycardia (n = 1 [1.0%]). Because postoperative AF represented most of the cases, the analyses focused on postoperative AF.

**Figure 1.**
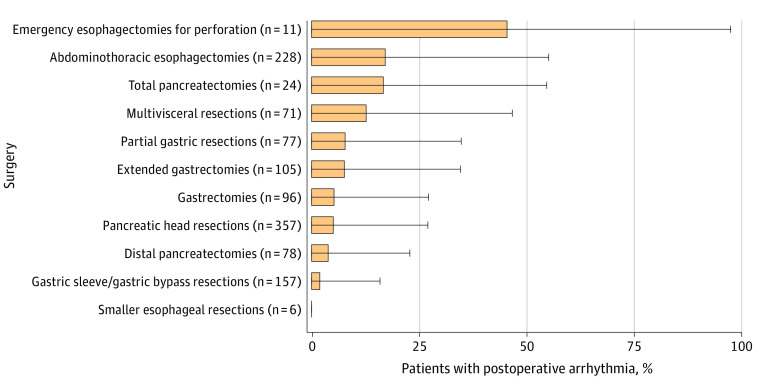
Incidence of Postoperative Atrial Fibrillation After Different Types of Upper Gastrointestinal Tract Surgery Error bars represent SDs of plus 1.

**Figure 2.**
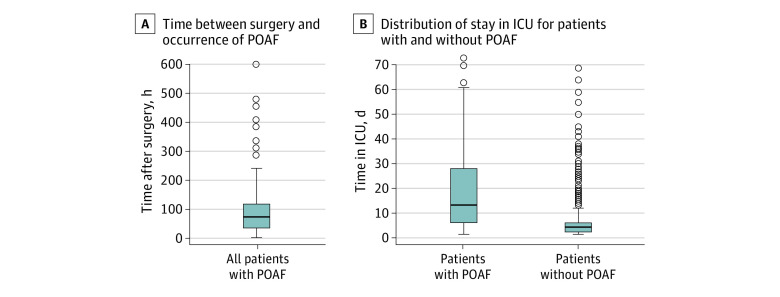
Time Between Surgery and Postoperative Atrial Fibrillation and Time Spent in Intensive Care Unit The center line in the box represents the median time, with the outer bounds of the box representing the range. Whiskers represent 95% CIs; circles, outliers; POAF, postoperative atrial fibrillation.

Overall, the incidence of postoperative AF was 8.3% (100 of 1210 patients) but varied considerably among different surgeries. The occurrence of postoperative AF was most frequently seen after complex esophageal resections (45.5%), followed by elective thoracoabdominal esophagectomies (17.1%) and total pancreatectomies (16.7%). The incidence of AF following multivisceral resections was 12.7%. Incidence of postoperative AF was 7.8% after partial gastric resections, 7.6% after extended gastrectomies, 5.2% after gastrectomies, 5.0% after pancreatic head resections, 1.9% after gastric sleeve or gastric bypass resections (representing minor surgeries compared with the others), 3.9% after distal pancreatectomies, and none after smaller esophageal resections. Figure 1 shows the incidence of postoperative AF among all the enrolled patients. The median time between surgery and postoperative AF was 72.0 (IQR, 36.5-112.8) hours (Figure 2A).

In total, 77 of 100 patients (77.0%) were treated with amiodarone, and 19 patients (19.0%) received electrical cardioversion; of these, 18 (18.8%) received both electric and medicated rhythm control. Rate control with β-blockers was performed in 33 patients (33.0%). In 10 patients (10.0%), spontaneous conversion into sinus rhythm occurred, and they did not receive medication for rhythm or rate control.

### Risk Factors for and Outcomes of Postoperative AF

**Figure 3.**
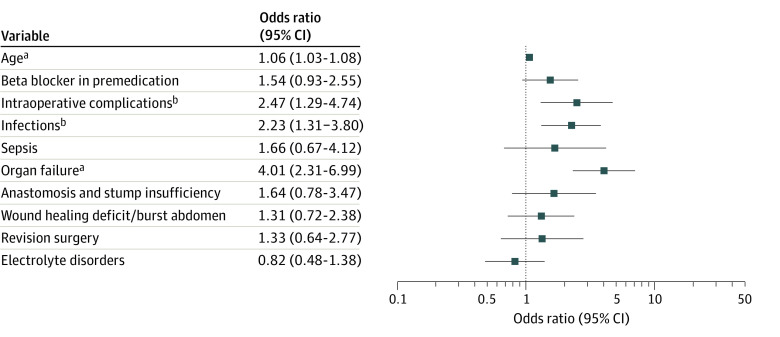
Variables Associated With Postoperative Atrial Fibrillation as Part of the Logistic Regression Model Whiskers represent the 95% CI. ^a^*P* < .001. ^b^*P* < .01.

In this analysis, the occurrence of postoperative AF was associated with older age (68.5 vs 60.2 years; *P* < .001; Figure 3). According to logistic regression analysis, 4 different variables were associated with the occurrence of postoperative AF: patients’ age (OR, 1.06; 95% CI, 1.03-1.08; *P* < .001), intraoperative surgical complications (OR, 2.47; 95% CI, 1.29-4.74; *P* = .006), infections (OR, 2.23; 95% CI, 1.31-3.80; *P* = .003), and organ failure (OR, 4.01; 95% CI, 2.31-6.99; *P* < .001; Figure 3).

**Figure 4.**
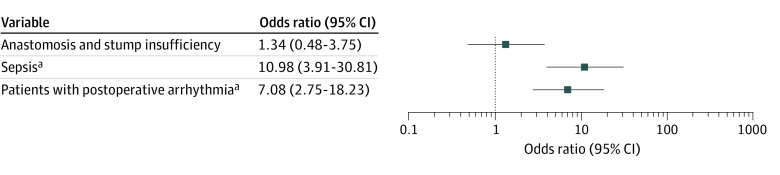
Variables of the Logistic Regression Model for In-Hospital Mortality Whiskers represent the 95% CI. ^a^*P* = .001.

Patients who developed postoperative AF had a significantly longer stay in the ICU than those without postoperative AF (23.4 days vs 5.9 days; *P* < .001; Figure 2B). Moreover, new-onset postoperative AF was independently associated with in-hospital mortality (OR, 7.08; 95% CI, 2.75-18.23; *P* < .001) and sepsis (OR, 10.98; 95% CI, 3.91-30.81; *P* < .001). Figure 4 shows the factors associated with in-hospital mortality.

**Figure 5.**
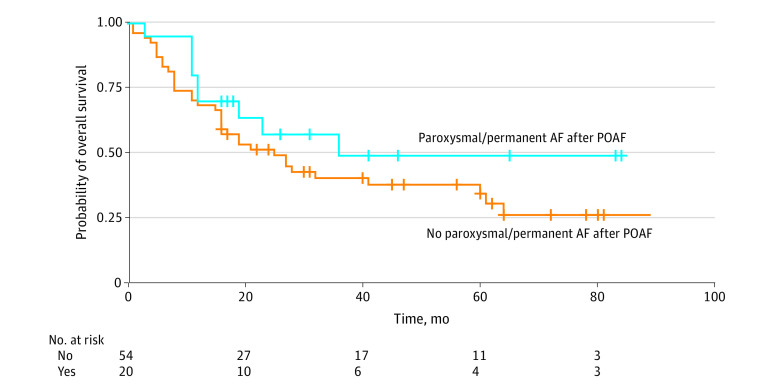
Kaplan-Meier Curve of Patients Who Did and Did Not Develop Paroxysmal or Permanent Atrial Fibrillation After Postoperative Atrial Fibrillation No significant difference in overall survival was observed.

Data from 74 of 100 patients (74.0%) were used for the follow-up. The remaining 26 patients (26.0%) who developed postoperative AF had either died in the hospital (n = 8 [8.0%]) or were lost to follow-up (n = 18 [18.0%]). The median follow-up time was 19 months. Altogether, 20 of 74 patients (27.0%) had developed persistent or paroxysmal AF. Compared with the 0.8% incidence of AF in an age-matched population,^13^ the incidence of development of permanent or paroxysmal AF after postoperative AF in the present study was significantly higher. However, regarding the overall survival data, no significant difference was observed between patients who developed a persistent or paroxysmal AF after postoperative AF and those who did not (Figure 5).

## Discussion

Analysis of the number of patients with postoperative AF after upper gastrointestinal surgery revealed a high number of arrhythmic events in patients undergoing esophageal resection. The 17.1% incidence of postoperative AF after elective esophageal surgery is similar to that of a meta-analysis by Schizas et al^21^ (16.5%). However, the reported incidence varies, ranging from 20.3% to 23.1%.^15,16,17^ Colwell et al^23^ reported a 39% incidence of AF after a series of transhiatal resections with transcervical endoscopic mobilization of the esophagus. However, a 1% incidence of AF after esophageal resection has been reported as well.^24^ Overall, it must be acknowledged that the types of cardiac arrhythmias or the technical type of documentation are different, as recently reported by Chebbout et al.^14^ Furthermore, Ojima et al^18^ found that the type of surgery or surgical reconstruction played a pivotal role in the incidence of postoperative AF. Other clinical parameters, such as preoperative therapy, may also be associated with AF after upper gastrointestinal surgery.^15^

In contrast to many existing studies, the focus of this study was to assess postoperative AF in elective thoracoabdominal esophagectomy as well as to understand its importance in visceral surgery of the upper gastrointestinal tract. Gastric and pancreatic surgical procedures were also analyzed. We aimed to group patients according to the expected complexity of the surgical procedure. As expected, the incidence of postoperative AF was higher in more intense surgical procedures (eg, multivisceral resections had a 12.7% incidence of postoperative AF). This finding became more evident in comparisons of more extended procedures vs less extended procedures in the same organ (eg, extended gastrectomy, including gastrectomy with distal transhiatal esophagectomy, had an 8.7% incidence of postoperative AF vs a 7.9% incidence with gastrectomy alone) or even extended gastrectomies vs gastric sleeve or gastric bypass resections (7.6% vs 1.9% incidence of postoperative AF). Similar results were found for total pancreatectomies vs pancreatic head resections (16.7% vs 5.0% incidence of postoperative AF).

Apart from the organ or complexity of the surgical procedures, preexisting inflammatory processes may be a trigger for postoperative AF. The group of patients with emergency esophageal resections had the highest incidence of postoperative AF (45.5%). This incidence was partly attributable to the esophageal resection itself; however, the inflammatory process in this area may have contributed as well. This inflammatory process might also explain the comparatively high incidence of postoperative AF in the group of patients who underwent partial gastric resections. Although it is a less complex procedure, the incidence of postoperative AF is similar to that of extended gastrectomy. Partial gastric resections are usually performed in an emergency setting when an ulcer is perforated or shows bleeding. We believe that 3 aspects of visceral surgery are relevant for the genesis of postoperative AF: (1) the organ the surgery is performed on, (2) the extent of the surgery, and (3) inflammatory aspects in emergency settings.

In the case of risk stratification, 2 additional aspects should be noted: older patients and those with previous β-blocker medication have a higher risk of developing postoperative AF. Age was associated with postoperative AF both in the present cohort and in those of other studies.^15,16,17,25^ In previous studies, hypertension was associated with a substantial risk of postoperative arrhythmia.^18,26^ Notably, previous treatment for cardiac disease was not associated with the occurrence of postoperative AF after correction for multiple testing, whereas history of cardiac disease, such as congestive heart failure, was previously described as a risk factor.^25^ The incidence of postoperative AF is associated with a higher number of postoperative complications, infections, and organ failure.

Previous analyses found that postoperative AF was not associated with increased in-hospital mortality,^16^ but our study revealed a significant association of postoperative AF with increased in-hospital mortality.

Although AF accounts for the highest number of cardiac arrhythmias, ventricular disorders also occur. It was not clear if these factors need to be considered separately. Because patients with these disorders also display cardiac dysregulation, there is a need for documentation and evaluation in the future. Furthermore, most patients in the present study developed postoperative AF 72 hours after the end of surgery (Figure 2A). A relevant number of undetected events of postoperative AF may occur if the postoperative surveillance period in the ICU is too short. In a study of long-term survival after esophagectomy, Wells et al^27^ found that postoperative AF was associated with worse long-term survival. Therefore, a more thorough cardiac observation after surgery is warranted in groups with risk factors for postoperative AF.

In the present study of 1210 patients, postoperative AF was associated with higher mortality and a prolonged postoperative ICU stay. Along with the development of sepsis, postoperative AF was shown to be an independent factor associated with increased in-hospital mortality (Figure 4).

Nevertheless, the association between postoperative AF and sepsis needs to be investigated along with the contribution of inflammation in triggering cardiac arrhythmia. However, these issues are beyond the scope of the present study and warrant future analyses using data from a prospective data registry. In addition, the question of whether the appearance of postoperative AF may serve as an early marker for complications in the immediate postoperative period warrants investigation.

As for long-term outcomes, a high proportion of patients with new-onset AF after discharge was observed: 27.0% of all patients with postoperative AF in the present study developed a permanent or paroxysmal AF, whereas the incidence of AF in a study with an age-matched population was 0.8%.^13^ The absence of an association between survival data and the development of permanent or paroxysmal AF after postoperative AF may be confounded by the poor prognosis of esophageal cancer and a high number of cancer-related deaths in general.

One possible method of preventing AF after cardiac surgery is the use of β-blockers, which was recommended in the latest European Society of Cardiology guidelines.^13^ In the present study, however, β-blocker medication was not associated with a reduction in the incidence of postoperative AF after visceral surgery.

### Limitations

This study has limitations. It is a retrospective study and therefore the object of possible bias. The association between sepsis and postoperative AF remains unclear. There is a possibility that postoperative AFs remain undetected when patients leave the ICU or intermediate care unit and are no longer monitored. Follow-up was possible in only 74.0% of patients who developed postoperative AF.

## Conclusions

The findings of the present study suggest that new-onset postoperative AF is associated with severe postoperative complications. Also, there are clinical aspects (age, organ failure, intraoperative complications) associated with postoperative AF. However, because of the retrospective nature of this study, possible interactions might remain undetected. We believe that the present study offers a solid foundation for future prospective clinical trials to ascertain if standardized preoperative screenings for AF and cardiological workup are helpful in patients with planned upper gastrointestinal surgery, especially in those with a high risk of postoperative AF (eg, older age, esophageal or extended surgery in general). Further analysis is needed regarding whether cardiac monitoring for at least 72 hours and consultation with cardiologists after discharge for patients with postoperative AF and risk factors, such as sepsis, infection, emergency surgery, extended surgery, and organ failure, would improve outcomes.

## Notes

Supplement. **eFigure 1.** Flowchart of Patient Enrollment **eFigure 2.** English Version of the Patient Survey **eTable 1.** Demographic and Clinical Data of All Enrolled Patients **eTable 2.** Survey Results at 1 Year of Follow-up
