# Supplementary material for: Incidence, Associated Risk Factors, and Outcomes of Postoperative Arrhythmia After Upper Gastrointestinal Surgery
Source: JAMA Netw Open. 2022 Jul 21;5(7):e2223225. doi: 10.1001/jamanetworkopen.2022.23225 (PMC9305375; doi:10.1001/jamanetworkopen.2022.23225)
Supplement: Supplement. — eFigure 1. Flowchart of Patient Enrollment eFigure 2. English Version of the Patient Survey eTable 1. Demographic and Clinical Data of All Enrolled Patients eTable 2. Survey Results at 1 Year of Follow-up [file jamanetwopen-e2223225-s001.pdf]

Supplementary Online Content

Rühlmann F, Tichelbäcker T, Mackert AF, et al. Incidence, associated risk factors, and outcomes of postoperative arrhythmia after upper gastrointestinal surgery. *JAMA Netw Open.* 5(7):e2223225. doi:10.1001/jamanetworkopen.2022.23225

eFigure 1. Flowchart of Patient Enrollment
eFigure 2. English Version of the Patient Survey
eTable 1. Demographic and Clinical Data of All Enrolled Patients
eTable 2. Survey Results at 1 Year of Follow-up

This supplementary material has been provided by the authors to give readers additional information about their work.

© 2022 Rühlmann F et al. *JAMA Network Open.*

eFigure 1. Flowchart of Patient Enrollment

Flow chart of patients‘ enrollement

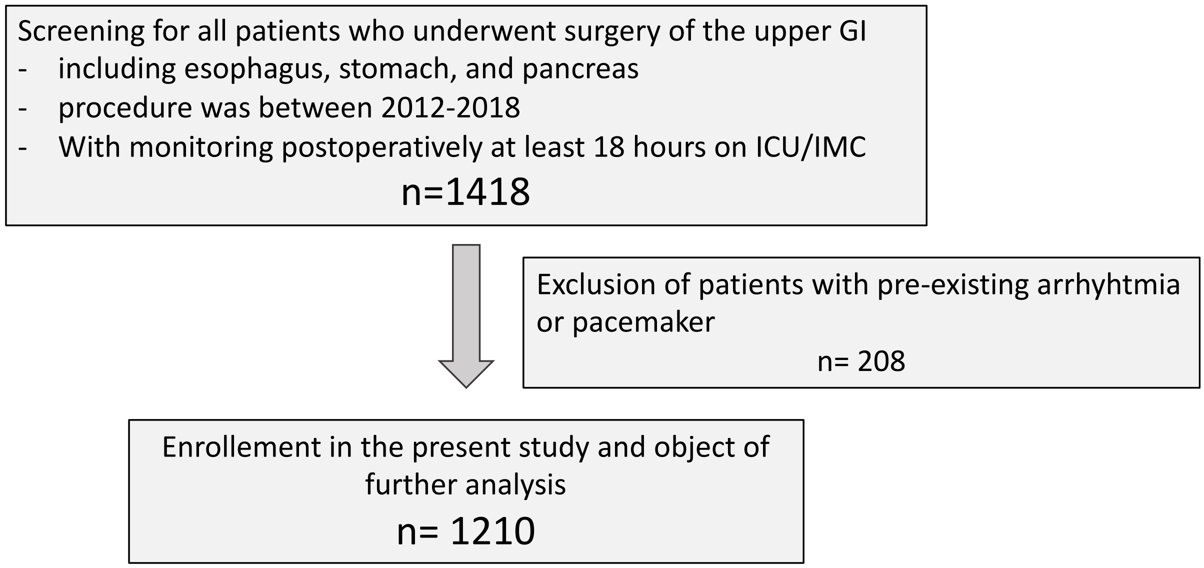

© 2022 Rühlmann F et al. *JAMA Network Open.*

eFigure 2. English Version of the Patient Survey

English version oft the patients‘ survey used

Universitätsmedizin Göttingen
Allgemein-, Viszeral- und Kinderchirurgie, Univ.-Prof. Dr. med. Michael Ghadimi
Robert-Koch-Str. 40, 37075 Göttingen

Klinik für Allgemein-, Viszeral- und Kinderchiru
Direktor: Univ.-Prof. Dr. med. Michael Ghac

Survey

37099 Göttingen **Brief**
Robert-Koch-Straße 40, 37075 Göttingen **Adr**
0551 39-66104 **Tel**
0551 39-66106
info@chirurgie-goettingen.de **E-**

Please mark applicable answer!

Göttingen, May 2019

1) Which year the surgery at UMG was performed?

2012	2013	2014	2015	2016	2017	2018

2) Which organ was the surgery performed on?

Esophagus	Gaster	Colon	Liver	Pancreas	Adrenal gland	other

3) Were any arrhythmia known before surgery?

Yes (type?)		No

4) Was any arrhythmia diagnosed after discharge from hospital (UMG)?

Yes (type?)		No

If yes, after what time period did the arrhythmia occur?

Still in hospital	During first 4 weeks after discharge	During first 3 months after discharge	During first 6 months after discharge	During first year after discharge	After fist year after discharge

5) Did you developed a stroke after discharge?

Ja		Nein

If yes, after what time period did the stroke occur?

Still in hospital	During first 4 weeks after discharge	During first 3 months after discharge	During first 6 months after discharge	During first year after discharge	After fist year after discharge

6) Do you take any blood-thinning medication?

Yes (what sort of medication?)		no

© 2022 Rühlmann F et al. *JAMA Network Open.*

eTable 1. Demographic and Clinical Data of All Enrolled Patients

parameter	level	total	male	female
n		1210	703	507
Age	mean ± sd	61 ± 14	62 ± 13	60 ± 15
	median (min; max)	62 (19; 91)	63 (19; 91)	62 (22; 88)
Malign diagnosis				
	No<br>Yes	398 (32.9%)<br>812 (67.1%)	170 (24.2%)<br>533 (75.8%)	288 (45%)<br>279 (55%)
Surgery				
	Salvage esophagectomies	11 (0.9%)	6 (0.9%)	5 (1.0%)
	Abdominothoracic esophagectomies	228 (18.8%)	183 (26.0%)	45 (8.9%)
	Total pancreatectomies	24 (2.0%)	8 (1.1%)	16 (3.2%)
	Multivisceral resections	71 (5.9%)	45 (6.4%)	26 (5.1%)
	Partial gastric resections	77 (6.4%)	39 (5.5%)	38 (7.5%)
	Extended gastrectomies	105 (8.7%)	74 (10.5%)	31 (6.1%)
	Gastrectomies	96 (7.9%)	57 (8.1%)	39 (7.7%)
	Pancreatic head resections	357 (29.5%)	203 (28.9%)	154 (30.4%)
	Distal pancreatectomies	78 (6.4%)	39 (5.5%)	39 (7.7%)
	Gastric sleeve/gastric bypass	157 (13.0%)	45 (6.4%)	112 (22.1%)
	Smaller esophageal resections	6 (0.5%)	4 (0.6%)	2 (0.4%)
Hypertension				
	No	669 (55.3%)	379 (53.9%)	290 (57.2%)
	Yes	541 (44.7%)	324 (46.1%)	217 (42.8%)
Diabetes mellitus				
	No	884 (73.1%)	517 (73.5%)	367 (72.4%)
	Yes (Type I/II)	185 (15.3%)	127 (18.1%)	58 (11.4%)
	Metabolic syndrome	141 (11.7%)	59 (8.4%)	82 (16.2%)
Venous thrombosis				
(preoperative)	No	1135 (93.8%)	653 (92.9%)	482 (95.1%)
	Yes	75 (6.2%)	50 (7.1%)	25 (4.9%)
Coronary heart disease				
	No	1046 (86.4%)	582 (82.8%)	464 (91.5%)
	Yes	164 (13.6%)	121 (17.2%)	43 (8.5%)
Embolic event				
	No	1128 (93.2%)	658 (93.6%)	470 (92.7%)
	Yes	82 (6.8%)	45 (6.4%)	37 (7.3%)
Myocardial infarction (preoperative)				
	No	1156 (95.5%)	661 (94.0%)	495 (97.6%)
	Yes	54 (4.5%)	42 (6.0%)	12 (2.4%)
Heart failure				

© 2022 Rühlmann F et al. *JAMA Network Open.*

	No	1168 (96.5%)	675 (96.0%)	493 (97.2%)
	Yes	42 (3.5%)	28 (4.0%)	14 (2.8%)
Valvular heart disease				
	No	1172 (96.9%)	679 (96.6%)	493 (97.2%)
	Yes	38 (3.1%)	24 (3.4%)	14 (2.8%)
Coagulopathies				
	No	1197 (98.9%)	700 (99.6%)	497 (98.0%)
	Yes	13 (1.1%)	3 (0.4%)	10 (2.0%)
Multiple cardiovascular diseases (≥2)				
	No	749 (61.9%)	438 (62.3%)	311 (61.3%)
	Yes	461 (38.1%)	265 (37.7%)	196 (38.7%)
Performed cardiac treatment				
	No<br>Yes	1110 (91,7%)<br>100 (8.3%)	626 (89.0%)<br>77 (11.0%)	484 (95.5%)<br>23 (4.5%)
Premedication				
	Anticoagulants	288 (23.8%)	190 (27.0%)	98 (19.3%)
	Antiarrhythmics	326 (26.9%)	177 (25.2%)	149 (29.4%)
	Diuretics	293 (24.2%)	163 (23.2%)	130 (25.6%)
	Ras-inhibitors	469 (38.8%)	270 (38.4%)	199 (39.3%)
	Oral antidiabetics	133 (11.0%)	80 (11.4%)	53 (10.5%)
Chirurgical complications intraoperative				
	No	1131 (93.5%)	650 (92.5%)	481 (94.9%)
	Yes	79 (6.5%)	53 (7.5%)	26 (5.1%)
Anastomosis-/ stump insufficiency				
	No	1089 (90.0%)	619 (88.1%)	470 (92.7%)
	Yes	121 (10.0%)	84 (11.9%)	37 (7.3%)
Wound healing deficit/ burst abdomen				
	No	1046 (86.4%)	595 (84.6%)	451 (89.0%)
	Yes	164 (13.6%)	108 (15.4%)	56 (11.0%)
Chyle/ pancreatic fistula, bile leakage				
	No	979 (80.9%)	574 (81.7%)	405 (79.9%)
	Yes	231 (19.1%)	129 (18,3%)	102 (20.1%)
Myocardial infarction (postoperative)				
	No	1206 (99.7%)	699 (99.4%)	507 (100.0%)
	Yes	4 (0.3%)	4 (0.6%)	0 (0.0%)
Revision surgery				
	No	1050 (86.8%)	594 (84.5%)	456 (89.9%)

© 2022 Rühlmann F et al. *JAMA Network Open.*

	Yes	160 (13.2%)	109 (15.5%)	51 (10.1%)
Organic failure				
	No	1095 (90.5%)	629 (89.5%)	466 (91.9%)
	Yes	115 (9.5%)	74 (10.5%)	41 (8.1%)
Electrolyte disorders				
	No	868 (71.7%)	502 (71.4%)	366 (72.2%)
	Yes	342 (28.3%)	201 (28.6%)	141 (27.8%)
Thrombosis				
(postoperative)				
	No	1194 (98.7%)	696 (99.0%)	498 (98.2%)
	Yes	16 (1.3%)	7 (1.0%)	9 (1.8%)
Infections				
	No	888 (73.4%)	498 (70.8%)	390 (76.9%)
	Yes	322 (26.6%)	205 (29.2%)	117 (23.1%)
Sepsis				
	No	1156 (95.5%)	669 (95.2%)	487 (96.1%)
	Yes	54 (4.5%)	34 (4.8%)	20 (3.9%)

© 2022 Rühlmann F et al. *JAMA Network Open.*

eTable 2. Survey Results at 1 Year of Follow-up

Paroxysmal/persistent arrhythmia after hospitalization	Number of events (dead) (n)	Number at risk (n)	Survival (n)	Survival in % (95% CI)
No (n=54)	17	38	37	68.5 (57.2-82.1)
Yes (n=20)	6	16	14	70 (52.5-93.3)

© 2022 Rühlmann F et al. *JAMA Network Open.*
